# White matter microstructure alterations in idiopathic restless legs syndrome: a study combining crossing fiber-based and tensor-based approaches

**DOI:** 10.3389/fnins.2023.1240929

**Published:** 2023-09-21

**Authors:** Yibo Xue, Sangma Xie, Xunheng Wang, Xugang Xi, Chunyan Liu

**Affiliations:** ^1^School of Automation, Hangzhou Dianzi University, Hangzhou, China; ^2^Department of Neurology, Xuanwu Hospital, Capital Medical University, Beijing, China; ^3^Beijing Key Laboratory of Neuromodulation, Beijing, China

**Keywords:** restless legs syndrome, white matter, diffusion MRI, partial volume fraction, tract-based spatial statistics, atlas-based analysis

## Abstract

**Introduction:**

Restless legs syndrome (RLS) is a common sensorimotor disorder characterized by an irrepressible urge to move the legs and frequently accompanied by unpleasant sensations in the legs. The pathophysiological mechanisms underlying RLS remain unclear, and RLS is hypothesized to be associated with alterations in white matter tracts.

**Methods:**

Diffusion MRI is a unique noninvasive method widely used to study white matter tracts in the human brain. Thus, diffusion-weighted images were acquired from 18 idiopathic RLS patients and 31 age- and sex-matched healthy controls (HCs). Whole brain tract-based spatial statistics (TBSS) and atlas-based analyzes combining crossing fiber-based metrics and tensor-based metrics were performed to investigate the white matter patterns in individuals with RLS.

**Results:**

TBSS analysis revealed significantly higher fractional anisotropy (FA) and partial volume fraction of primary (F1) fiber populations in multiple tracts associated with the sensorimotor network in patients with RLS than in HCs. In the atlas based analysis, the bilateral anterior thalamus radiation, bilateral corticospinal tract, bilateral inferior fronto-occipital fasciculus, left hippocampal cingulum, left inferior longitudinal fasciculus, and left uncinate fasciculus showed significantl increased F1, but only the left hippocampal cingulum showed significantly higher FA.

**Discussion:**

The results demonstrated that F1 identified extensive alterations in white matter tracts compared with FA and confirmed the hypothesis that crossing fiber-based metrics are more sensitive than tensor-based metrics in detecting white matter abnormalities in RLS. The present findings provide evidence that the increased F1 metric observed in sensorimotor tracts may be a critical neural substrate of RLS, enhancing our understanding of the underlying pathological changes.

## 1. Introduction

Restless legs syndrome (RLS), which includes idiopathic RLS, is a prevalent neurological disorder characterized by an uncontrollable urge to move the legs, frequently accompanied by uncomfortable sensations. This condition mainly affects patients in evenings and nights, leading to sleep disruption and a significant impact on quality of life (Allen et al., 2003; Trenkwalder et al., 2005; Scholz, 2017). Recent epidemiological analyzes in different countries have determined that the RLS prevalence rate ranges from 3.9 to 15% in the general population (Ohayon et al., 2012). Despite the prevalence and influence of RLS, its fundamental mechanisms are still not entirely understood. The potential pathophysiology of RLS involves dysfunction in the dopaminergic system, iron deficiency, and alterations in central nervous system excitability (Allen, 2015; Guo et al., 2017). A comprehensive understanding of the pathogenesis of RLS is essential for prompt diagnosis and treatment, which can alleviate symptoms and improve the quality of life of patients. Recently, an increasing number of magnetic resonance imaging (MRI) studies employing various modalities have provided evidence implicating the microstructures of white matter tracts and structural connectivity in the pathophysiology of RLS (Provini and Chiaro, 2015; Rizzo and Plazzi, 2018; Aldemir et al., 2020; Kassubek, 2022). Advanced neuroimaging applications to RLS can provide information useful to improve the understanding of its pathophysiology (Rizzo and Plazzi, 2018).

Diffusion MRI (dMRI) is a noninvasive imaging technique that can be employed to examine the white matter microstructure of the live human brain (Tournier, 2019). Diffusion MRI is widely used in connectivity and network analysis since it probes the diffusion of water molecules to uncover details about neural microstructure (Zuo et al., 2012; Xie et al., 2015, 2016). Various diffusion MRI studies have employed diffusion tensor imaging (DTI) to investigate white matter tracts in patients with RLS through voxel-based or tract-based methods, although the results have exhibited considerable heterogeneity among studies (Unrath et al., 2008; Rizzo et al., 2012; Chang et al., 2014; Belke et al., 2015; Lindemann et al., 2016; Zhuo et al., 2017; Rizzo and Plazzi, 2018; De Paiva et al., 2020; Park et al., 2021). Previous studies have reported significant changes in tract-related alterations in the corpus callosum, thalamic radiation, sensorimotor areas, sensorimotor white matter, frontal gyrus white matter, primary and associated motor white matter and limbic/nociceptive networks (Rizzo and Plazzi, 2018). An early DTI study found significantly altered fractional anisotropy (FA), obtained from a tensor-based approach, in the bilateral primary and associated motor and somatosensory cortices (Unrath et al., 2008). Furthermore, DTI studies have shown abnormal patterns in the corpus callosum, internal capsule, white matter in the inferior frontal gyrus, and temporal regions among patients with RLS (Chang et al., 2014; Belke et al., 2015). Nevertheless, some DTI studies have failed to detect any significant differences in white matter between patients with RLS and healthy controls (Rizzo et al., 2012; Zhuo et al., 2017). Recently, a graph theory-based DTI study examined the brain network topology of patients with RLS and discovered significant differences in both global and local structural connectivity (Park et al., 2022). Considering the pivotal role of dMRI in studying white matter tracts and the heterogeneity of findings from previous dMRI-based studies, further investigation of the structural connectivity in patients with RLS using dMRI is essential to advancing its pathophysiology.

Currently, region-of-interest (ROI) analysis and voxel-based analysis are the commonly employed analytical methods in dMRI studies of RLS brain connectivity. Nonetheless, white matter atlas-based ROI analysis approaches are more specific than traditional ROI-based analysis (Grinberg et al., 2017). Compared to voxel-based analysis (VBA) methods, tract-based spatial statistics (TBSS) can reduce the impact of partial volume effects, coregistration errors, and structural changes to a certain extent (Smith et al., 2006). Another limitation of previous studies is the use of fractional anisotropy (FA), a tensor-based measure, as a metric (Rizzo and Plazzi, 2018). The tensor-based approach assumes the presence of a single coherently orientated fiber population within a voxel (Tuch et al., 2002). However, this assumption is not always valid, and multiple fiber populations can be detected in over 90% of imaging voxels (Jeurissen et al., 2013). Tensor-derived metrics, such as FA, may be insensitive to changes in white matter microstructure and ambiguous when interpreting those changes (Beaulieu, 2002). The crossing-fiber approach estimates partial volume fractions for different fiber orientations, including primary (F1) and secondary (F2) orientations, and provides more accurate interpretation within regions of complex architecture compared to traditional tensor-based methods (Behrens et al., 2007; Baumgartner et al., 2015). The utilization of F1 and F2 metrics hopes to associate local changes in white matter with particular fiber population, thereby making the interpretation of white matter fiber abnormalities more tract specific. An increase in F1 values suggest increased relative amount of the primary fiber population within a voxel (Jbabdi et al., 2010). TBSS using metrics derived from the crossing-fiber approach, specifically F1 and F2, has been employed in recent studies to investigate properties of white matter and is suggested to improve the comprehensibility of the results (Morie et al., 2017; Yip et al., 2017; Seitz-Holland et al., 2021). However, to the best of our knowledge, it has not been employed in RLS studies.

Therefore, the aim of the current study was to investigate the different patterns of white matter microstructure in patients with RLS compared with healthy controls using two distinct methods, whole brain TBSS and atlas-based averaging over 20 anatomically defined white matter (WM) regions. In addition, to investigate potential pathological mechanisms in RLS, FA derived from the traditional tensor-based method, as well as partial volume fractions of primary (F1) and secondary (F2) fiber populations quantified by a crossing fiber-based model (Behrens et al., 2007; Jbabdi et al., 2010), were utilized in these two methods. Based on the results of previous dMRI studies, we hypothesized that alterations of multiple white matter tracts in RLS will be examined and crossing fiber-based metrics would offer increased sensitivity for detecting WM microstructural changes and identifying more extensive alterations in comparison to tensor-based metric.

## 2. Materials and methods

### 2.1. Participants

The current study included 18 right-handed, idiopathic RLS patients and 31 age- and sex-matched right-handed healthy controls (HCs). RLS patients were recruited from outpatient clinics at Xuan Wu Hospital and diagnosed by a sleep medicine neurologist based on the International Restless Legs Syndrome Study Group (IRLSSG) criteria (Allen et al., 2003) through clinical interviews. HCs were recruited by advertisement from the local community. Patients’ symptom severity was assessed by using the IRLSSG Rating Scale and the Johns Hopkins Restless Legs Severity Scale. In addition, sleep quality was evaluated for all patients with the Pittsburgh Sleep Quality Index (PSQI). Patients were further assessed on the Hamilton Anxiety Rating Scale (HAM-A) and Hamilton Depression Rating Scale (HAM-D) to identify and exclude those with scores greater than 21 and 20, respectively, which indicate severe anxiety or depression. Individuals with a history of alcohol or drug abuse, anemia, renal disease, spinal cord or nerve root injury, neuropathies, or other sleep disorders were excluded from the study. In all the 18 patients, 15 patients are drug-naïve, and three patients had a history of dopaminergic medication use. Out of the three patients, two took pramipexole at a daily dose of 0.125 mg for 12 months, while the remaining patient took the same medication and dose for a duration of 3 months. The study protocol was approved by the Medical Research Ethics Committee at Xuan Wu Hospital of Capital Medical University, and all the participants provided written informed consent before the study.

### 2.2. Magnetic resonance imaging data acquisition and processing

MRI data were collected on a 3.0 T Siemens Trio Scanner with an 8-channel SENSE head coil. The diffusion-weighted imaging (DWI) data were obtained through a single-shot spin-echo echo-planar imaging sequence with the following parameters: field-of-view (FOV) was 256 mm × 256 mm, matrix size was 128 × 128 (128 × 116 for 18 participants), in-plane resolution was 2 mm × 2 mm, thickness was 2 mm with no slice gap, with 70 slices (60 slices for 18 participants) collected. The repetition time (TR) was 7,000 ms, echo time (TE) was 91 ms, and flip angle was 90°. For each subject, five volumes without diffusion weighting (*b* = 0 s/mm^2^, b0) and 30 volumes with diffusion weighting (*b* = 1,000 s/mm^2^) along 30 gradient directions were collected.

Two specialists visually inspected the images to identify any artifacts resulting from image acquisition. To correct the distortions in the diffusion weighted imaging (DWI) data induced by head motion and eddy currents, we applied the eddy_correct function, *eddy_correct*, in FSL 5.0 (Jenkinson et al., 2012). The *fdt_rotate_bvecs* function in FSL (Leemans and Jones, 2009) was then utilized to adjust the diffusion gradient directions according to the transformation applied during eddy current correction. To create brain mask images for the DWI data, skull stripping was applied (Smith, 2002).

After the above corrections and rotation, the FA maps were calculated using the FSL FDT tool with a tensor-based approach (Jenkinson et al., 2012). Bayesian Estimation of Diffusion Parameters Obtained using Sampling Techniques for Crossing Fibers (BedpostX) was employed to establish distribution on diffusion parameters at each voxel (Behrens et al., 2003, 2007). This method provides voxelwise modeling of multiple fiber orientations and yields partial volume estimates (PVEs) specific to each orientation with a crossing fiber-based approach. Specifically, BedpostX provides PVEs for the primary and secondary fiber orientations (F1 and F2, respectively) of each voxel (Behrens et al., 2003, 2007; Jbabdi et al., 2010; Arkesteijn et al., 2020).

### 2.3. Tract-based spatial statistics and atlas-based analysis

TBSS analysis based on crossing-fiber measurements first requires a full TBSS analysis using FA images (Jbabdi et al., 2010). Therefore, voxelwise statistical analysis of the FA data was carried out using TBSS in FSL (Smith et al., 2006). First, all subjects’ FA data were then aligned into a 1 × 1 × 1 mm^3^ FMRIB58 FA standard space using the nonlinear registration tool FNIRT (Jenkinson et al., 2012), which uses a b-spline representation of the registration warp field (Rueckert et al., 1999). Next, the mean FA image was created and thinned to create a mean FA skeleton that represented the centers of all tracts common to the group. Voxels with FA values greater than 0.2 on the skeleton were selected for statistical analysis to exclude interference from gray matter and cerebrospinal fluid. Then, PVEs corresponding to the primary (F1) and secondary (F2) fiber orientations for each voxel were integrated into the tract-based spatial statistics analyzes using tbss_x (Jbabdi et al., 2010). tbss_x automatically ensures that a fiber orientation x1 in a given subject corresponds to the same fiber population across all subjects and applies nonlinear warps and skeleton projection to the crossing fiber measurements (Jbabdi et al., 2010). Finally, each subject’s aligned FA, F1, and F2 data were projected onto this skeleton for voxelwise cross-subject statistics.

The atlas-based analysis employed the Johns Hopkins University (JHU) White Matter Atlas, which is available in FSL software and comprises 20 white matter tracts. The tracts included the left/right anterior thalamic radiation (ATR_L/R), left/right corticospinal tract (CST_L/R), left/right cingulate gyrus part of the cingulum (Cg_L/R), left/right hippocampal part of the cingulum (Ch_L/R), forceps major (FMA), forceps minor (FMI), left/right inferior fronto-occipital fasciculus (IFOF_L/R), left/right inferior longitudinal fasciculus (ILF_L/R), left/right superior longitudinal fasciculus (SLF_L/R), left/right uncinate fasciculus (UF_L/R), and left/right superior longitudinal fasciculus temporal (SLFt_L/R). Each subject’s FA, F1, and F2 images were aligned to standard space in TBSS analysis. The intersection region of the skeleton and the tract region obtained from the JHU white-matter atlas was defined as the core region of the tract. Finally, average FA/F1/F2 scalar metrics of the core regions were derived for the 20 tracts.

### 2.4. Statistical analysis

Statistical analyzes were performed using IBM SPSS version 22.0 (IBM Corporation, 2020). The demographic characteristics of patients and healthy controls were compared using an independent-samples *t*-test for age and a chi-square test for sex.

Voxelwise statistical analysis was performed across subjects using the “*randomize*” tool, which is a permutation-based inference tool for nonparametric statistics implemented in FSL (Nichols and Holmes, 2002; Winkler et al., 2014). Group comparisons between individuals with RLS and healthy controls were performed using a general linear model that included age, sex, and acquisition parameters as covariates of no interest. The mean FA skeleton, with a threshold of 0.2, was used as a mask for the analysis, which involved 50,000 permutations. To correct for multiple comparisons, the significance threshold was determined with a *p* < 0.05 (two-tailed) after correcting for family-wise error (FWE) using the threshold-free cluster enhancement (TFCE) option in FSL (Smith and Nichols, 2009). To improve the visualization of significant differences, the *tbss_fill* script, which is implemented in FSL, was used to thicken the corresponding regions. We employed the “cluster” command in FSL to generate corresponding clusters of statistically significant regions and report their information. The fiber tracts corresponding to statistically significant regions were located by utilizing the “*atlasquery”* command along with Johns Hopkins University’s ICBM-DTI-81 atlas provided by the FSL. To quantitatively compare the ability of F1/F2 indices and conventional FA indices to detect abnormalities in white matter fibers, we computed the proportion of abnormal voxels in the whole skeleton mask voxels for each index.

For atlas-based analysis, general linear model (GLM) analyzes were performed using SPSS, with mean FA/F1/F2 values of each tract region as the dependent variable, group (RLS patients and healthy controls) as the independent variable, and age, sex and scan parameters as variables of no interest. The statistical significance of the result was determined using a threshold of *p* < 0.05 (FWE corrected for multiple tests across 20 tract regions).

## 3. Results

### 3.1. Demographic characteristics

The demographic and clinical characteristics of RLS patients and HCs are presented in Table 1. There were no significant differences in age or sex ratio between the two groups (*p* > 0.05). All the subjects included in the study were right-handed.

**Table 1:** Demographic and clinical characteristics of the participants.

Characteristic	HC	RLS	Statistics
	*n* = 31	*n* = 18	
Age (years)	59.97 (7.11)	57.67 (8.50)	*t* = 1.016, *p* = 0.315
Sex (male:female)	11:20	6:12	*χ*^2^ = 0.023, *p* = 0.879
Handedness (right/left)	31:0	18:0	NA
Duration of illness (years)	NA	21.11 (11.79)	NA
IRLSSG scores	NA	26.39 (6.20)	NA
PSQI scores	NA	11.39 (3.51)	NA
HAM-D scores	NA	5.33 (2.26)	NA
HAM-A scores	NA	9.06 (3.42)	NA

Means and standard deviations are reported unless otherwise specified. HC, healthy control; RLS, restless legs syndrome; NA, not applicable; IRLSSG, International Restless Legs Syndrome Study Group; PSQI, Pittsburgh Sleep Quality Index; HAM-D, Hamilton Depression Rating Scale; HAM-A, Hamilton Anxiety Rating Scale.

### 3.2. Tract-based spatial statistics analysis

**Figure 1:**
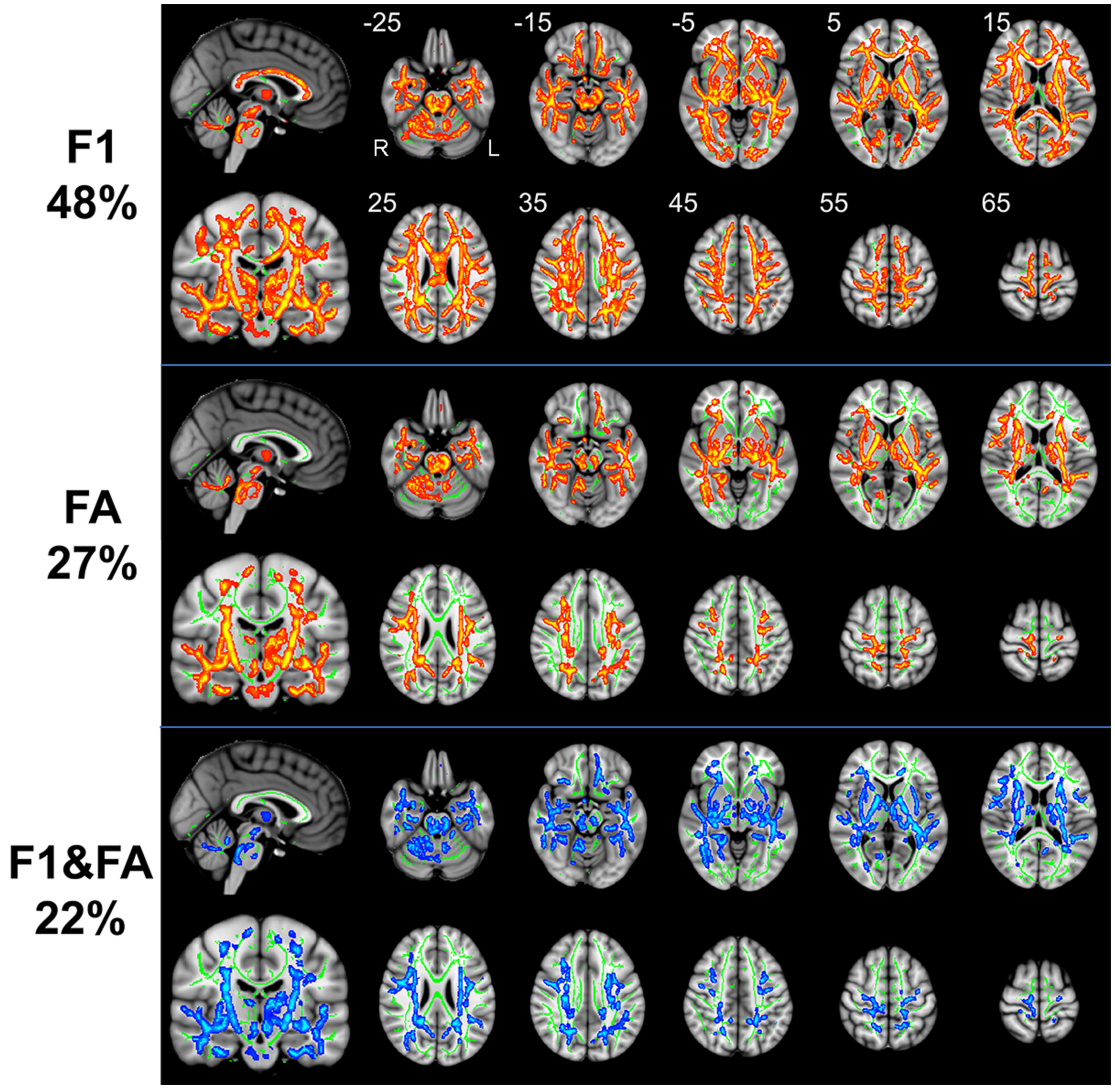
TBSS analysis revealed white matter regions with significant differences in F1 and FA values between RLS patients and healthy controls (*p* < 0.05, FWE corrected). Green represents the mean FA skeleton of all participants; red denotes an increase in RLS patients compared with healthy controls; blue denotes the intersection regions of areas with significant differences in F1 and FA metrics. The percentage in the left column represents the percentage of the abnormal voxels relative to the whole skeleton voxels for each metric.

The results of whole-brain TBSS analysis on the FA metric obtained with the tensor-based approach and the F1/F2 metrics obtained with the crossing fiber-based approach are shown in Figure 1.

#### 3.2.1. Crossing fiber-based approach

Compared to healthy controls, patients with RLS exhibited significantly increased F1 value (*p* < 0.05, FWE corrected), which is the PVE corresponding to the primary fiber orientation, in multiple white matter regions with complex fiber configurations (Figure 1). Moreover, this between-group difference was observed extensively throughout the whole brain white matter skeleton (Figure 1). Fiber tract regions with significantly higher F1 value, identified by the “*atlasquery”* command along with JHU White-Matter Tractography Atlas, primarily consisted of the forceps minor, bilateral SLF, bilateral IFOF, bilateral ILF, bilateral ATR, bilateral CST, left UF, and left cingulum. There were no significant group differences in the F2 values of the white matter skeleton as determined by the TBSS analysis. Clusters showing significantly higher F1 value in RLS patients compared with HCs in TBSS analysis were illustrated in Table 2. The regions with significant differences formed into a single large cluster containing 69,873 voxels and the minimal corrected *p* value was 0.003.

**Table 2:** Clusters showing significantly higher F1 value in RLS patients compared with HCs in TBSS analysis.

Cluster index	Cluster voxels	Peak MNI coordinate			*P* value (minimum)	Tracts
		*x*	*y*	*z*		
1	69,873	45	156	50	0.003	Forceps minor, bilateral SLF, bilateral IFOF, bilateral ILF, bilateral ATR, bilateral CST, left UF, and left cingulum.

RLS, restless legs syndrome; HCs, healthy controls; TBSS, tract-based spatial statistics; MNI, Montreal Neurological Institute; SLF, superior longitudinal fasciculus; IFOF, inferior fronto-occipital fasciculus; ILF, inferior longitudinal fasciculus; ATR, anterior thalamic radiation; CST, corticospinal tract; UF, uncinate fasciculus.

#### 3.2.2. Tensor-based approach

Patients with RLS, compared to healthy controls, demonstrated significantly higher FA values in some regions of the white matter skeleton (*p* < 0.05, FWE corrected, Figure 1). However, the area with significant differences was smaller than that identified in terms of F1 values. Fiber tract regions with significantly higher FA values, identified by the “*atlasquery”* command along with JHU White-Matter Tractography Atlas, primarily consisted of the forceps minor, bilateral SLF, bilateral IFOF, bilateral ILF, bilateral CST, bilateral ATR, and left cingulum. Clusters showing significantly higher FA value in RLS patients compared with HCs in TBSS analysis were illustrated in Table 3. The regions with significant differences formed into three clusters and Cluster 3&2 (including 21,145 and 18,007 voxels respectively) were much larger than Cluster 1 (including 290 voxels).

**Table 3:** Clusters showing significantly higher FA value in RLS patients compared with HCs in TBSS analysis.

Cluster index	Cluster voxels	Peak MNI coordinate			*P* value (minimum)	Tracts
		*x*	*y*	*z*		
3	21,145	120	98	78	0.015	Left SLF, left IFOF, left ILF, left ATR, left CST, forceps minor, left cingulum
2	18,007	63	99	69	0.014	Right IFOF, right ILF, right SLF, right ATR, right CST
1	290	83	121	75	0.045	Right ATR

FA, fractional anisotropy; RLS, restless legs syndrome; HCs, healthy controls; TBSS, tract-based spatial statistics; MNI, Montreal Neurological Institute; SLF, superior longitudinal fasciculus; IFOF, inferior fronto-occipital fasciculus; ILF, inferior longitudinal fasciculus; ATR, anterior thalamic radiation; CST, corticospinal tract.

#### 3.2.3. F1 vs. FA

**Figure 2:**
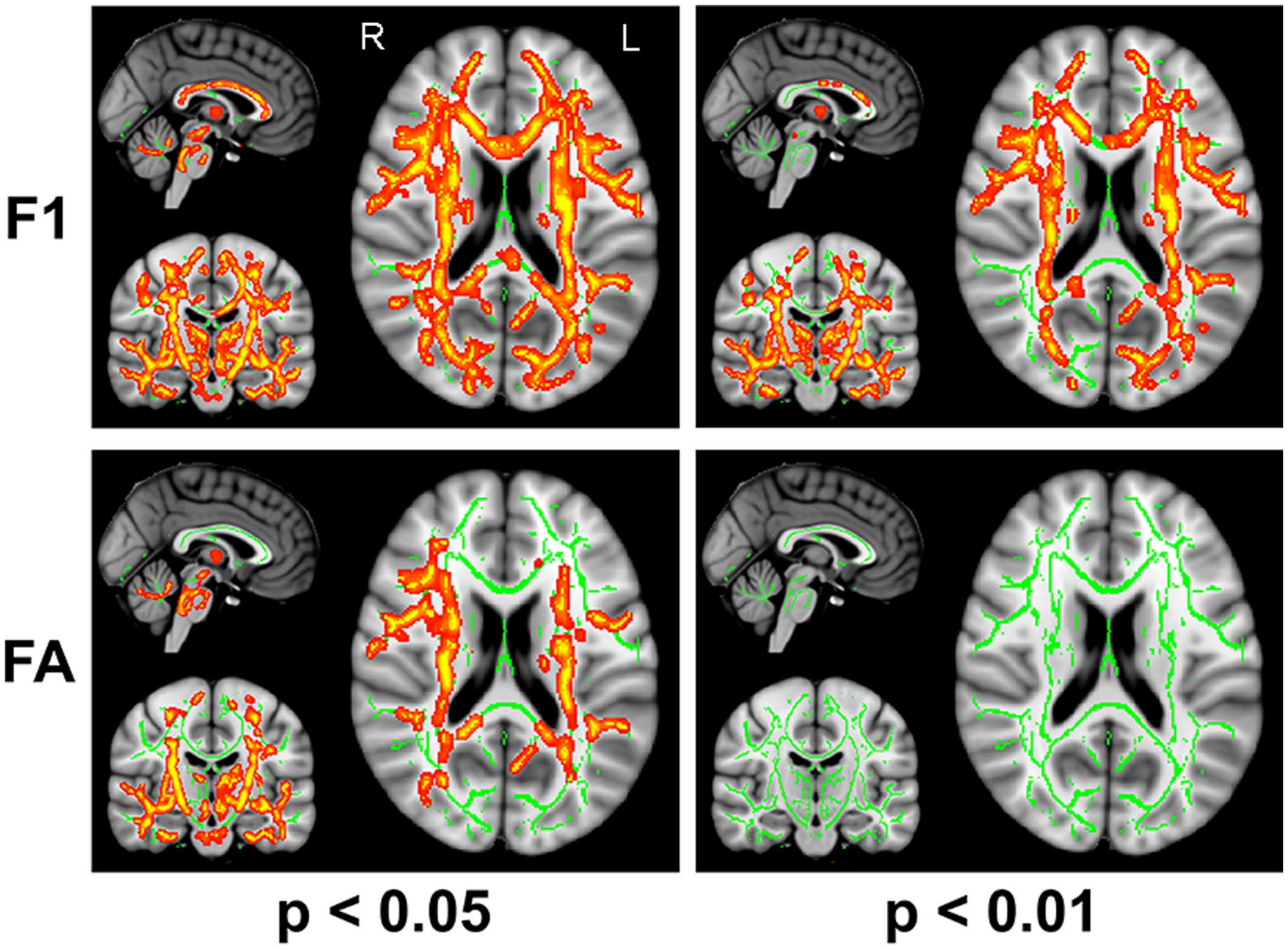
TBSS analysis identified white matter regions with significant differences in F1 and FA values between RLS patients and healthy controls using two different significance thresholds (*p* < 0.05 and *p* < 0.01, FWE corrected). Green represents the mean FA skeleton of all participants; red denotes an increase in RLS patients compared with healthy controls.

The F1 values from the crossing fiber-based approach and the FA values from the tensor-based approach detected abnormal patterns in 48 and 27% of voxels of the whole white matter skeleton, respectively. The intersection of areas with significant differences in F1 and FA metrics contained 22% of voxels of the whole white matter skeleton (Figure 1), indicating that the majority of voxels exhibiting differences in FA values were within the area exhibiting a significant difference in F1 metrics. The intersection of areas primarily consisted of the forceps minor, bilateral SLF, bilateral IFOF, bilateral ILF, bilateral ATR, bilateral CST, and left UF. The areas with a between-group difference in F1 values in the white matter skeleton were larger than those with a between-group difference in FA values, as shown in Figures 1, 2, which display regions with the same significance threshold for F1 and FA values. The abnormal pattern of the F1 metrics survived when the *p* value threshold was as low as *p* < 0.01 and was highly consistent with the pattern observed at a p value of 0.05 (Figure 2). In contrast, the corresponding patterns of FA differences at a significance level of 0.05 did not survive at such a stringent threshold (*p* < 0.01, FWE corrected; Figure 2).

### 3.3. Atlas-based WM analysis

#### 3.3.1. Crossing fiber-based analysis

**Figure 3:**
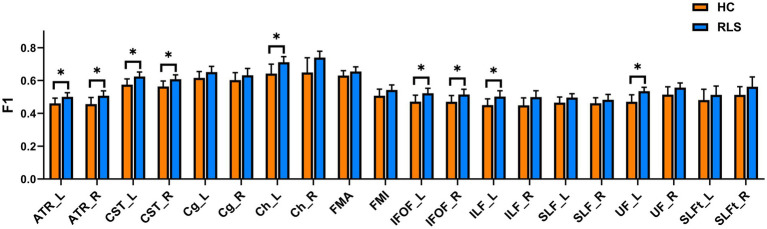
Mean values with standard deviations of the F1 metrics in RLS patients and HCs for the 20 tracts. Significant group differences were identified using a general linear model analysis, where * indicates a statistically significant difference at *p* < 0.05 with FWE correction.

The group comparison of F1 metrics for the anatomical ROIs (20 WM tracts) is shown in Figure 3. The results indicate that patients with RLS had significantly higher F1 values in the bilateral ATR, bilateral CST, bilateral IFOF, left hippocampal cingulum (Ch), left ILF, and left UF than HCs (*p* < 0.05, FWE corrected). Groupwise differences between healthy controls and restless legs syndrome patients for F1 within all the 20 white matter tracts were reported in Supplementary Table S1.

#### 3.3.2. Tensor-based approach

**Figure 4:**
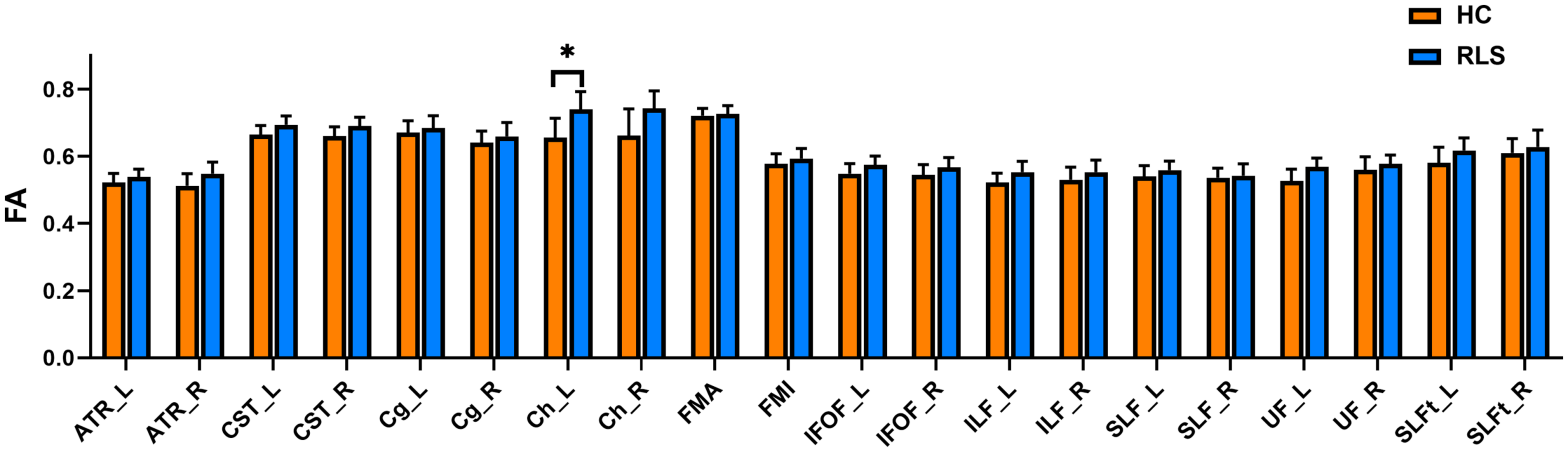
Mean values with standard deviations of the FA metrics in RLS patients and HCs for the 20 tracts. Significant group differences were identified using a general linear model analysis, where * indicates a statistically significant difference at *p* < 0.05 with FWE correction.

The group comparison of FA metrics for the anatomical ROIs (20 WM tracts) is shown in Figure 4. The results indicate that patients with RLS had significantly higher FA values than HCs in only the left Ch (*p* < 0.05, FWE corrected). Although the FA values of the bilateral ATR, bilateral CST bilateral IFOF, left ILF, and left UF in patients were slightly higher than those in HCs, the differences were not significant. Groupwise differences between healthy controls and restless legs syndrome patients for FA within all the 20 white matter tracts were reported in Supplementary Table S1.

## 4. Discussion

In this study, partial volume fractions of multiple fiber populations (F1 and F2) and FA values from a conventional tensor-based approach were examined to identify alterations in white matter throughout the brain of patients with RLS, with the use of TBSS analysis and atlas-based analysis. The findings demonstrate the utility of combining crossing fiber-based metrics and conventional tensor-based metrics in the assessment of microstructural changes in white matter; extensive white matter impairments were found in RLS patients with these two kinds of metrics. Compared with the healthy control group, patients with RLS symptoms showed significantly higher F1 and FA values in the tracts connected to the frontal gyrus (including the forceps minor, SLF, IFOF, ATR, and CST) as well as in the ILF. Furthermore, the crossing fiber-based metrics were more sensitive than FA values for detecting abnormalities in white matter regions with different fiber arrangements.

We discovered that using tensor-based FA indicators in patients with RLS revealed changes of distinct regions of white matter fiber bundles consistent with the majority of DTI studies on RLS (Unrath et al., 2008; Chang et al., 2014; Lindemann et al., 2016; Rizzo and Plazzi, 2018; Park et al., 2022). Additionally, this study is the first to use a crossing fiber-based F1 index for studying RLS, and we identified a wider range of affected fiber bundle regions. With the significance threshold of p < 0.05, a high degree of overlap was observed between the regions with significant differences in fiber bundles detected with the F1 metrics and FA metric in the TBSS analysis. However, with application of a more stringent threshold (*p* < 0.01), the FA index was unable to identify significant differences in white matter fiber bundles. In the atlas-based analysis, the F1 metrics exhibited superior efficacy in detecting abnormalities in white matter fibers compared to the FA metric, which detected significant differences in only the left hippocampal cingulum. The altered white matter tracts identified by F1 in the TBSS analysis were highly consistent with those detected in the atlas-based analysis. These altered tracts included the bilateral ATR, CST, and IFOF, which are associated with the sensorimotor network (Catani and Thiebaut de Schotten, 2008; Radwan et al., 2022) and have been implicated in previous dMRI studies of RLS (Unrath et al., 2008; Chang et al., 2014; Belke et al., 2015). The current study showed that the F1 metrics based on the crossing-fiber model is more sensitive than the FA metric in examining abnormal patterns in white matter regions containing complex fiber structures. In addition, the performance of the F1 metrics in detecting white matter abnormalities in studies of other brain diseases (Morie et al., 2017; Yip et al., 2017; Park et al., 2021) was consistent with the findings of this study.

In TBSS analysis, a considerable number of various fiber bundle regions were detected by the FA index possessing significant differences. However, the FA values of most fiber bundles did not exhibit any significant differences in ROI-based analysis. This result can be attributed to the unique analytical characteristics of the TBSS and ROI-based methods (Van Hecke et al., 2016). In the ROI-based analysis, information of individual voxel or sub-region might be lost as diffusion measures are averaged over the whole white matter tract region. TBSS evaluates and compares diffusion measures at the most precise imaging scale possible: the individual voxel (Smith et al., 2006). As such, the obtained diffusion measures have the potential of being more sensitive as well as specific. Given the strengths, weaknesses, and underlying assumptions associated with different analysis techniques, we employed a combination of TBSS analysis and ROI-based analysis in our study. The F1 values revealed much higher significance of the between-group differences in both atlas-based and TBSS analysis even at a relatively small number of subjects. This finding is important in the context of the minimum group size and statistical reliability required for investigation (Szczepankiewicz et al., 2013).

The forceps minor, left SLF, bilateral IFOF, bilateral ILF, bilateral ATR, bilateral CST, and left cingulum were among the altered tracts identified repeatedly in the TBSS analysis using crossing fiber-based metrics and tensor-based metrics. The CST originates from the motor cortex, specifically the primary motor cortex and the supplementary motor area, which are key components of the sensorimotor network. It is a major fiber pathway that plays a crucial role in motor control and the transmission of sensory information (Jang, 2009). The SLF is a fiber bundle connecting the prefrontal, parietal, and temporal lobes and is responsible for regulating motion and processing visual–spatial information (Makris et al., 2005). Studies have shown that disruptions or lesions in the cingulum and ATR can result in various motor and sensory abnormalities (Nelson, 2021; Rashidi et al., 2023). The bilateral IFOF comprises a pair of fiber tracts that connect cortical areas between the frontal and occipital lobes. Although the principal function of the IFOF primarily relates to visual and spatial cognition, it is involved in the sensorimotor network (Sarubbo et al., 2013). The abnormal fiber tracts identified in our study were mainly distributed within the sensorimotor network, which is consistent with the fiber tracts that may be associated with the uncontrollable urge to move the legs, a clinical symptom associated with RLS. These findings provide evidence of potential pathophysiological alterations in central nervous system excitability.

The altered tracts identified in this study had increased FA values with higher F1 values, which indicates hyperconnectivity of white matter, in RLS patients compared to healthy controls. However, Unrath et al. (2008) reported decreased FA values in close proximity to the primary and associate motor and somatosensory cortices. Belke et al. (2015) reported both increased and decreased FA values in temporal regions. Rizzo and Plazzi (2018) found no differences between patients with RLS and healthy controls using TBSS. The lack of biological specificity of FA might be one possible reason for these conflicting results. FA can be affected by various factors, such as axonal integrity, myelination, tissue density, and packing, among others (Le Bihan and Johansen-Berg, 2012). A decrease in FA values can result from either a decrease in the relative amount of the primary fiber population or an increase in the relative amount of the secondary fiber population. Additionally, simultaneous increases or decreases in the relative amount of fibers in both directions (primary and secondary) may not result in a significant alteration in FA values. Therefore, we applied more specific metrics, the partial volume fractions of primary (F1) and secondary (F2) fiber populations, to dissect the abnormal patterns of white matter. Patients with RLS exhibited a notable increase in the F1 metric, while no significant changes in specific regions were observed for the F2 metric. This pattern suggested an increase in the relative amount of the primary fiber population in those areas, while the relative amount of the secondary fiber population remained unchanged. The significantly higher F1 observed in patients with RLS aligns with the findings of elevated FA values and provides evidence of the pathophysiology of central nervous system excitability in RLS.

This study has several limitations that should be addressed in future work. First, to guarantee an adequate sample size, three patients had a history of dopaminergic medication use, which might affect white matter integrity (Van Der Schaaf et al., 2013), and patients without any history of drug or physical therapy were included in the study. Second, the sample size was relatively small, which might limit its statistical power. In addition, the inability of FA values to detect abnormal fibers in the ROI analysis may also be associated with the small sample size. Therefore, future studies should recruit drug-naive patients to mitigate the impact of treatment-related confounding variables and improve the statistical power. Third, the two methods used in our study only allowed us to identify the abnormal tracts, but an advanced tract segmentation method, i.e., automated fiber quantification (Yeatman et al., 2012), could identify the specific segment along the tract that is altered in patients with RLS. Finally, more specific metrics, including the mean kurtosis (obtained from diffusion kurtosis imaging) (Jensen et al., 2005) and neurite density imaging (obtained from neurite orientation dispersion and density imaging) (Zhang et al., 2012), should be employed to reveal the underlying mechanism of white matter alteration in RLS.

In conclusion, this study showed the potential effectiveness of combining crossing fiber-based metrics with conventional tensor-based metrics to assess microstructural alterations in white matter. Our results demonstrated extensive alterations in the white matter tracts associated with the sensorimotor network in RLS. The increased FA values observed in the tracts may result from an increase in the partial volume fractions of primary (F1) fiber populations. TBSS analysis and atlas-based analysis showed that group differences in crossing fiber-based metrics were more widespread than those in tensor-based metrics. The current study confirmed that combining tensor-based and crossing-fiber-based metrics provided additional, specific, and complementary information regarding white matter properties in RLS. These findings suggest that F1 metrics can serve as a sensitive neuroimaging endophenotype of white matter microstructural impairments in RLS and contribute to a better understanding of the pathophysiology of RLS.

## Data availability statement

The raw data supporting the conclusions of this article will be made available by the authors, without undue reservation.

## Ethics statement

The studies involving humans were approved by Medical Research Ethics Committee at Xuan Wu Hospital of Capital Medical University. The studies were conducted in accordance with the local legislation and institutional requirements. The participants provided their written informed consent to participate in this study.

## Author contributions

YX was responsible for the design of the study, the analysis of the MRI data, and the preparation of the manuscript. SX was responsible for the design of the study, the analysis of the MRI data, the interpretation of the findings, and the preparation of the manuscript. XW was responsible for the analysis of the MRI data and the preparation of the manuscript. XX was responsible for the design of the study and the preparation of the manuscript. CL was responsible for the design of the study, obtaining ethical approval, collecting participants and MRI data, the interpretation of the findings, and the preparation of the manuscript. All authors contributed to the article and approved the submitted version.
