# Supplementary material for: White matter microstructure alterations in idiopathic restless legs syndrome: a study combining crossing fiber-based and tensor-based approaches
Source: Front Neurosci. 2023 Sep 21;17:1240929. doi: 10.3389/fnins.2023.1240929 (PMC10551141; doi:10.3389/fnins.2023.1240929)
Supplement: Supplementary file 1 [file Table_1.DOCX]

# Supplementary Materials for

White Matter Microstructure Alterations in Idiopathic Restless Legs Syndrome: A Study Combining Crossing Fiber-based and Tensor-based Approaches

**Tables:**

**Table S1**. Groupwise differences between healthy controls and restless legs syndrome patients for F1 and FA within the 20 white matter tracts.

| Tracts | F1 | | | | FA | | | |
| --- | --- | --- | --- | --- | --- | --- | --- | --- |
|  | Mean (Standard) | | P value | Corrected P | Mean (Standard) | | P value | Corrected P |
|  | HC | RLS |  |  | HC | RLS |  |  |
| ATR_L | 0.4611 (0.0320) | 0.5010 (0.0254) | <0.0001 | 0.0020 | 0.5221 (0.0271) | 0.5387 (0.0234) | 0.0051 | 0.1018 |
| ATR_R | 0.4562 (0.0406) | 0.0254 (0.0303) | 0.0007 | 0.0136 | 0.5128 (0.0356) | 0.5487 (0.0341) | 0.0061 | 0.1218 |
| CST_L | 0.5750 (0.0355) | 0.6246 (0.0274) | 0.0012 | 0.0243 | 0.6646 (0.0274) | 0.6945 (0.0255) | 0.0105 | 0.2095 |
| CST_R | 0.5636 (0.0342) | 0.6079 (0.0268) | 0.0021 | 0.0423 | 0.6607 (0.0276) | 0.6907 (0.0258) | 0.0106 | 0.2150 |
| Cg_L | 0.6162 (0.0392) | 0.6514 (0.0349) | 0.0402 | 0.8041 | 0.6705 (0.0353) | 0.6843 (0.0368) | 0.3168 | 1.0000 |
| Cg_R | 0.6026 (0.0455) | 0.6320 (0.0419) | 0.0415 | 0.8290 | 0.6406 (0.0346) | 0.6595 (0.0417) | 0.1176 | 1.0000 |
| Ch_L | 0.6424 (0.0578) | 0.7111 (0.0350) | <0.0001 | 0.0004 | 0.6556 (0.0583) | 0.7402 (0.0531) | <0.0001 | 0.0003 |
| Ch_R | 0.6492 (0.0902) | 0.7399 (0.0387) | 0.0036 | 0.0727 | 0.6618 (0.0796) | 0.7425 (0.0530) | 0.0095 | 0.1896 |
| FMA | 0.6301 (0.0306) | 0.6552 (0.0281) | 0.1483 | 1.0000 | 0.7213 (0.0218) | 0.7260 (0.0253) | 0.9127 | 1.0000 |
| FMI | 0.5072 (0.0404) | 0.5427 (0.0306) | 0.0110 | 0.2197 | 0.5780 (0.0300) | 0.5934 (0.0299) | 0.1486 | 1.0000 |
| IFOF_L | 0.4713 (0.0396) | 0.5227 (0.0303) | 0.0016 | 0.0330 | 0.5486 (0.0302) | 0.5751 (0.0257) | 0.0254 | 0.5088 |
| IFOF_R | 0.4704 (0.0388) | 0.5151 (0.0323) | 0.0018 | 0.0354 | 0.5452 (0.0307) | 0.5676 (0.0290) | 0.0242 | 0.4840 |
| ILF_L | 0.4502 (0.0382) | 0.5022 (0.0356) | 0.0010 | 0.0200 | 0.5223 (0.0281) | 0.5531 (0.0323) | 0.0113 | 0.2265 |
| ILF_R | 0.4492 (0.0457) | 0.4993 (0.0389) | 0.0030 | 0.0609 | 0.5308 (0.0371) | 0.5534 (0.0359) | 0.0486 | 0.9720 |
| SLF_L | 0.4655 (0.0340) | 0.4959 (0.0243) | 0.0247 | 0.4945 | 0.5403 (0.0324) | 0.5591 (0.0272) | 0.1313 | 1.0000 |
| SLF_R | 0.4619 (0.0335) | 0.4827 (0.0332) | 0.4289 | 1.0000 | 0.5363 (0.0292) | 0.5425 (0.0351) | 0.9017 | 1.0000 |
| UF_L | 0.4708 (0.0424) | 0.5350 (0.0244) | 0.0006 | 0.0122 | 0.5269 (0.0353) | 0.5690 (0.0263) | 0.0066 | 0.1310 |
| UF_R | 0.5140 (0.0480) | 0.5568 (0.0289) | 0.0558 | 1.000 | 0.5600 (0.0391) | 0.5778 (0.0262) | 0.1778 | 1.0000 |
| SLFt_L | 0.4811 (0.0659) | 0.5128 (0.0536) | 0.4102 | 1.0000 | 0.5811 (0.0460) | 0.6174 (0.0376) | 0.2034 | 1.0000 |
| SLFt_R | 0.5127 (0.0499) | 0.5621 (0.0603) | 0.0105 | 0.2100 | 0.6099 (0.0432) | 0.6285 (0.0501) | 0.1517 | 1.0000 |

FA, fractional anisotropy; HC, healthy control; RLS, restless legs syndrome; ATR_L/R, left/right anterior thalamic radiation; CST_L/R, left/right corticospinal tract; Cg_L/R, left/right cingulate gyrus part of the cingulum; Ch_L/R, left/right hippocampal part of the cingulum; FMA, forceps major; FMI, forceps minor; IFOF_L/R, left/right inferior fronto-occipital fasciculus; ILF_L/R, left/right inferior longitudinal fasciculus; SLF_L/R, left/right superior longitudinal fasciculus; UF_L/R, left/right uncinate fasciculus; SLFt_L/R, left/right superior longitudinal fasciculus temporal.
